# The Influence of Second-Hand Cigarette Smoke Exposure during Childhood and Active Cigarette Smoking on Crohn’s Disease Phenotype Defined by the Montreal Classification Scheme in a Western Cape Population, South Africa

**DOI:** 10.1371/journal.pone.0139597

**Published:** 2015-09-30

**Authors:** Tawanda Chivese, Tonya M. Esterhuizen, Abigail Raffner Basson

**Affiliations:** 1 Community Health Division, Department of Interdisciplinary Health Sciences, University of Stellenbosch, Cape Town, South Africa; 2 Centre for Evidence-Based Health Care, Department of Interdisciplinary Health Sciences, University of Stellenbosch, Cape Town, South Africa; 3 Department of Dietetics, University of the Western Cape, Cape Town, South Africa; CWRU/UH Digestive Health Institute, UNITED STATES

## Abstract

**Background:**

Smoking may worsen the disease outcomes in patients with Crohn’s disease (CD), however the effect of exposure to second-hand cigarette smoke during childhood is unclear. In South Africa, no such literature exists. The aim of this study was to investigate whether disease phenotype, at time of diagnosis of CD, was associated with exposure to second-hand cigarette during childhood and active cigarette smoking habits.

**Methods:**

A cross sectional examination of all consecutive CD patients seen during the period September 2011-January 2013 at 2 large inflammatory bowel disease centers in the Western Cape, South Africa was performed. Data were collected via review of patient case notes, interviewer-administered questionnaire and clinical examination by the attending gastroenterologist. Disease phenotype (behavior and location) was evaluated at time of diagnosis, according to the Montreal Classification scheme. In addition, disease behavior was stratified as ‘complicated’ or ‘uncomplicated’, using predefined definitions. Passive cigarette smoke exposure was evaluated during 3 age intervals: 0–5, 6–10, and 11–18 years.

**Results:**

One hundred and ninety four CD patients were identified. Cigarette smoking during the 6 months prior to, or at time of diagnosis was significantly associated with ileo-colonic (L3) disease (RRR = 3.63; 95%CI, 1.32–9.98, p = 0.012) and ileal (L1) disease (RRR = 3.54; 95%CI, 1.06–11.83, p = 0.040) compared with colonic disease. In smokers, childhood passive cigarette smoke exposure during the 0–5 years age interval was significantly associated with ileo-colonic CD location (RRR = 21.3; 95%CI, 1.16–391.55, p = 0.040). No significant association between smoking habits and disease behavior at diagnosis, whether defined by the Montreal scheme, or stratified as ‘complicated’ vs ‘uncomplicated’, was observed.

**Conclusion:**

Smoking habits were associated with ileo-colonic (L3) and ileal (L1) disease at time of diagnosis in a South African cohort.

## Introduction

Crohn’s disease (CD), a subtype of inflammatory bowel disease (IBD), is a heterogeneous disorder, which results from a deregulated host immune response. CD aetiology is associated with both genetic and environmental factors [1,2].

Cigarette smoking has been implicated in the development of complicated and aggressive CD over time [3–6]. While the mechanism(s) underlying the pathogenic role of smoking in CD remain unclear, a number of theories have been forwarded. These include; the up-regulation of susceptibility genes due to direct interaction with cigarette products; repeated irritation of intestinal mucosal T-cells by nicotine; and the consequent increased production of pro-inflammatory cytokines such as tumor necrosis factor alpha (TNFα), an important mediator in the inflammatory cascade [1,7]. In addition, cigarette smoking has been associated with imbalances in the gut microbiome [8], as well as exerting disruptive effects on RNA post translation methylation [9].

A wealth of literature originating from Western Europe and North America have consistently demonstrated an association between cigarette smoking and risk of future CD development, increased disease severity and reduced response to therapy [10–14]. In contrast, recent studies from Israel [15–17], China [18] and Asia [19], have failed to identify an association, implying differences in genetic susceptibilities or environmental factors, as well as the related interactions. Furthermore, discrepancies have been found in European studies too [3]. Moreover, of the few epidemiological observations investigating the effect of second-hand cigarette smoke exposure during childhood, findings for many have been contradictory [10,20,21]. It is possible that these discrepancies are attributed to heterogeneous study designs, differences in disease classification methods, or methodological challenges (e.g. recall bias, level of exposure), although CD susceptibility mutations may also influence outcomes.

The majority of people in South Africa are broadly categorized into three racial groups; Black South Africans, White and Coloured South Africans [22]. Notably, significant differences in the allele and genotype frequencies of CD susceptibility mutations have been identified between these three racial groups [23–25]. A recent South African study [26] reported South African Coloured CD patients to be significantly more likely to develop ‘complicated’ CD (60% vs 9%, p = 0.023), when compared to their White counterparts. In a second report, the same authors identified a significant risk-association between second-hand cigarette smoke exposure during childhood (0–5 and 11–18 years) and risk of future CD development. Moreover, an exposure to second-hand cigarette smoke during the age interval 11–18 years, was found to be independently associated with CD risk [27].

The aim of this study was to investigate whether disease phenotype (location and behavior), at time of diagnosis of CD was associated with exposure to second-hand (‘passive’) cigarette during childhood and active cigarette smoking habits, in the above mentioned cohort.

## Materials and Methods

### Design and Setting

This was a cross-sectional study (part of a larger case-control study), performed between September 2011 and January 2013 of all consecutive CD patients age 18–70 years, seen during their normally scheduled appointments at, the 2 largest referral based IBD clinics in the Western Cape, South Africa; Groote Schuur Hospital (GSH) and Tygerberg Hospital (TBH). Details of the study cohort and study methodology have been described in detail elsewhere [26,27]. Briefly, disease diagnosis was confirmed according to the European Crohn’s and Colitis Organization (ECCO) guidelines [2]. The Montreal classification scheme [2] was used to define CD phenotype. Complicated disease was defined as the presence of any one of the following at diagnosis; fistulizing CD, stricturing CD, perianal fistulas or surgical resection. Only patients with complete data at diagnosis were included. Patients with a disease duration of less than 5 years, and those with a prior diagnosis of amoebiasis or tuberculosis, were excluded.

### Data Collection and Variables

Following informed consent, an investigator-administered questionnaire was used to collect data on; 1) participant demographics; 2) cigarette smoking habits during the 6 months prior to, or at the time of diagnosis; and 3) second-hand (‘passive’) cigarette smoke exposure pertaining to 3 age intervals during childhood; 0–5, 6–10 and 11–18 years. Passive cigarette smoke exposure was defined as having a smoker living permanently and smoking regularly inside the home. Patients were stratified either as a ‘smoker’ or ‘non-smoker’, according to reported smoking history. A ‘smoker’ was defined as smoking at least 7 cigarettes per day for at least 6 months.

### Ethical Considerations

Ethical approval for the main study, including the current research was granted by the Senate Ethics Committee of the University of the Western Cape (Reg. no. 11/3/16), the Human Ethics Research Committee of the University of Cape Town (Ref. no. 122/2011), and the Western Cape Provincial Department of Health. For all participants written informed consent was provided prior to study enrolment in the larger study.

### Statistical Analysis

Stata 12 [28] was used for analysis. Continuous data were tested for normality, and Student’s t-tests were used to test hypotheses. A 95% confidence interval was used for estimation and the level of significance was set at p < 0.05. For proportions, the Chi-squared and Fisher’s exact test were used, as appropriate, to test hypotheses.

Multivariate logistic regression analysis was used to adjust for confounders using three models. The first model used multinomial logistic regression with CD behaviour as a categorical outcome with inflammatory CD (B1) set as the reference outcome and adjusted for gender, ethnicity and CD location at time of diagnosis. The second model, used multinomial logistic regression with CD location as a categorical outcome and colonic (L2) disease set as the reference outcome. Confounders adjusted for included age, age at diagnosis, gender, ethnicity, duration of symptom onset until initial CD diagnosis as well as CD behaviour at time of diagnosis. Adjusted relative risk ratios (RRR) are reported for the multinomial logistic regression models. A third model used binary logistic regression with complicated CD as the outcome and adjusted for gender, ethnicity and CD location at time of diagnosis. In further analysis multinomial regression was carried out for the effect of passive cigarette smoke exposure in the smokers and non-smokers, sub-grouped. CD location and CD behavior confounded each other, and were adjusted for in each of the 3 models as appropriate.

## Results

Over a period of approximately 17 months, 194 CD patients meeting our inclusion criteria were identified. The demographic and baseline characteristics of the CD patients are shown in Table 1. Overall, 141 (73%) of the cohort were female and 53 (27%) were male. One hundred and forty three (74%) CD patients were ‘smokers’ during the 6 months prior to diagnosis, or at time of diagnosis. In addition, 144 (74%) CD patients were exposed to passive cigarette smoke during the age interval 0–5 years, 148 (76%) were exposed during 6–10 years, and 154(79%) were exposed during the age interval 11–18 years.

**Table 1:** Demographic and baseline characteristics of participants.

Characteristic		CD patients (N = 194)
Age at enrollment (median and IQR, yr.) *		47(38.0–57.0)
Symptom duration before CD diagnosis (median and IQR, months)		25.3(19–34)
Gender, no. (%)		
	Female	141 (72.7)
	Male	53 (27.3)
Ethnicity, no. (%)		
	Coloured	152 (78.4)
	White	35 (18.0)
	Black	7 (3.6)
Education, no. (%)†	Tertiary	36 (18.5)
Monthly income, no. (%)		
	<R10 000	191 (98.5)
	>R10 000	3 (1.6)
Smokers, no. (%)‡		143(73.3)
Passive cigarette smoke exposure during three age intervals, yr., no. (%)		
	0–5	144 (74.2)
	6–10	148 (76.3)
	11–18	154 (79.4)
Cumulative passive cigarette smoke exposure, yr., no. (%)		
	During 2 age intervals only	14 (7.2)
	During all 3 age intervals	133 (68.6)
Any passive cigarette smoke exposure, no. (%)		158 (81.4)

*Data missing for one subject.

^†^ At least some tertiary education.

^‡^ Cigarette smoking during the 6 months prior to diagnosis or at time of diagnosis.

### Smoking Exposure and Disease Phenotype, Defined by the Montreal Scheme

#### Disease Behavior

There was no significant difference between the proportions of patients with inflammatory (B1), stricturing (B2) and penetrating (B3) disease who were exposed to childhood passive cigarette smoke, during the 3 age intervals, respectively [(0–5 years: 79.1% vs 73.8% vs 63.8%, p = 0.223); (6–10 years: 78.1% vs 81.0% vs 68.1%, p = 0.360); and (11–18 years: 81.9% vs 83.3% vs 70.2%, p = 0.969)], when compared to those who were not exposed (Table 2). The risk of stricturing (B2) disease, compared to risk of inflammatory (B1) disease was higher in patients who were smokers during the 6 months prior to, or at the time of diagnosis, when compared to non-smokers, although the association was not significant (RRR = 2.50; 95%CI, 0.86–7.29, p = 0.093) (Table 2). In further analysis, childhood passive cigarette smoke exposure was not significantly associated with CD disease behaviour when smokers and non-smokers were sub-grouped and analysed separately (Table 2).

**Table 2:** Cigarette smoking and CD behavior according to the Montreal Classification.

					Stricturing vs inflammatory		Penetrating vs inflammatory	
		Inflammatory	Stricturing	Penetrating	Multivariate analysis*		Multivariate analysis*	
		n(%)	n(%)	n(%)	RRR^†^ (95%CI)	P-value	RRR^†^ (95%CI)	P-value
Overall								
Passive 0–5 yrs	Yes	83 (79.1)	31 (73.8)	30 (63.8)	0.30 (0.04–2.07)	0.223	0.40 (0.06–2.68)	0.344
	No	19 (18.1)	9 (21.4)	15 (31.9)				
	Missing	3 (2.9)	2 (4.8)	2 (4.3)				
Passive 6–10 yrs	Yes	82 (78.1)	34 (81.0)	32 (68.1)	2.70 (0.32–22.44)	0.360	0.61 (0.21–12.22)	0.643
	No	20 (19.1)	8 (19.1)	15 (31.9)				
	Missing	3 (2.9)	0	0				
Passive 11–18 yrs	Yes	86 (81.9)	35 (83.3)	33 (70.2)	1.03 (0.26–4.13)	0.969	0.58 (0.18–1.92)	0.373
	No	19 (18.1)	7 (16.7)	14 (29.8)				
Smoking^‡^	Yes	75 (71.4)	36 (85.7)	32 (68.1)	2.50 (0.86–7.29)	0.093	1.12 (0.49–2.61)	0.781
	No	29 (27.6)	5 (11.9)	12 (25.5)				
	Missing	1 (1.0)	1 (2.4)	3 (6.4)				
Non-smokers								
Passive 0–5 yrs	Yes	23 (79.3)	3 (60.0)	6 (50.0)	0.33 (0.02–4.68)	0.414	0.39 (0.08–1.99)	0.260
	No	6 (20.7)	2 (40.0)	5 (41.7)				
	Missing	0	0	1 (8.3)				
Passive 6–10 yrs	Yes	22 (75.9)	3 (60.0)	7 (58.3)	0.35 (0.03–4.92)	0.437	0.46(0.09–2.28)	0.345
	No	7 (24.1)	2 (40.0)	5 (41.7)				
	Missing	0	0	0				
Passive 11–18 yrs	Yes	20 (69.0)	3 (60.0)	8 (66.7)	0.68 (0.10–4.77)	0.899	0.90 (0.21–3.78)	0.886
	No	9 (31.0)	2 (40.0)	4 (33.3)				
Smokers								
Passive 0–5 yrs	Yes	59 (78.7)	28 (77.8)	21 (65.6)	0.28 (0.02–3.43)	0.316	0.49 (0.04–6.19)	0.585
	No	13 (17.3)	6 (16.7)	10 (31.3)				
	Missing	3 (4.0)	2 (5.6)	1 (3.1)				
Passive 6–10 yrs	Yes	59 (78.7)	31 (86.1)	22 (68.8)	12.71(0.65–248.39)	0.094	1.43(0.10–20.78)	0.792
	No	13 (17.1)	5 (13.9)	10 (31.2)				
	Missing	3 (4.0)	0	0				
Passive 11–18 yrs	Yes	65 (86.7)	31 (86.1)	22 (68.8)	0.50 (0.07–3.35)	0.472	0.42 (0.07–2.64)	0.356
	No	10 (13.3)	5 (13.9)	10 (31.2)				

Inflammatory (B1), Stricturing (B2), Penetrating (B3).

*Adjusted for gender, ethnicity, age at diagnosis and symptom duration before CD diagnosis and CD location at diagnosis.

^†^ Relative risk ratio, in comparison to risk of inflammatory disease.

^‡^ Smoking during 6 months prior to, or at time of diagnosis.

#### Disease location

In the multinomial logistic regression model, patients who smoked prior to, or at time of diagnosis, compared to non-smokers had a significantly higher risk of ileo-colonic (L3) disease (RRR = 3.63; 95%CI, 1.32–9.98, p = 0.013) and ileal (L1) disease (RRR = 3.54; 95%CI, 1.06–11.83, p = 0.040) compared with risk of colonic disease. Overally, no significant association between passive cigarette smoke exposure during the 3 age intervals 0–5, 6–10 or 11–18 years, was observed (Table 3). However, when smokers were analysed separately, childhood passive cigarette smoke exposure during the 0–5 years age interval was significantly associated ileo-colonic CD location (RRR = 21.3; 95%CI, 1.16–391.55, p = 0.040). The wide 95% confidence interval is a consequence of the reduced sample size in the sub-group analysis. In non-smokers no significant association was found between childhood passive cigarette smoke exposure and CD location (Table 3).

**Table 3:** Cigarette smoking and CD location according to the Montreal Classification.

					Ileal vs colonic as base outcome		Ileo-colonic vs colonic	
					Multivariate analysis*		Multivariate analysis*	
		Colonic n(%)	Ileal n(%)	Ileo-colonic n(%)	RRR^‡^ (95%CI)	P-value	RRR^‡^ (95%CI)	P-value
Overall								
Passive 0–5 yrs	Yes	24 (64.9)	31 (79.5)	87 (75.0)	13.10 (0.84–203.63)	0.066	6.32 (0.72–55.48)	0.096
	No	11 (29.7)	8 (20.5)	24 (20.7)				
	Missing	2 (5.4)	0	5 (4.3)				
Passive 6–10 yrs	Yes	25 (67.6)	30 (79.9)	91 (78.5)	0.13 (0.01–2.18)	0.157	0.39 (0.04–4.08)	0.433
	No	10 (27.0)	9 (23.1)	24 (20.7)				
	Missing	2 (5.4)	0	1 (0.9)				
Passive 11–18 yrs	Yes	29 (78.4)	31 (79.5)	92 (79.3)	0.36 (0.56–2.26)	0.274	0.26 (0.05–1.26)	0.094
	No	8 (21.6)	8 (20.5)	24 (20.7)				
Smoking†	Yes	23 (62.2)	31 (76.9)	92 (75.9)	3.54 (1.06–11.83)	0.040	3.63 (1.32–9.98)	0.013
	No	14 (37.8)	9 (23.1)	23 (19.8)				
	Missing	0	0	5 (4.3)				
Non-smokers								
Passive 0–5 yrs	Yes	8 (57.1)	7 (77.8)	17 (73.9)	2.19 (0.32–15.03)	0.427	1.77 (0.41–7.58)	0.422
	No	5 (35.7)	2 (22.2)	6 (26.7)				
	Missing	1 (7.1)	0	0				
Passive 6–10 yrs	Yes	9 (64.3)	6 (66.7)	17 (73.9)	1.11 (0.19–6.49)	0.907	1.57 (0.37–6.61)	0.536
	No	5 (35.7)	3 (33.3)	6 (26.1)				
Passive 11–18 yrs	Yes	11 (78.6)	5 (55.6)	15 (65.2)	0.34 (0.05–2.13)	0.250	0.51 (0.11–2.38)	0.393
	No	3 (21.4)	4 (44.4)	8 (34.8)				
Smokers								
Passive 0–5 yrs	Yes	16 (69.6)	24 (80.0)	66 (75.0)	14.9 (0.59–371.91)	0.100	21.3 (1.16–391.55)	0.040
	No	6 (25.1)	6 (20.0)	17 (19.3)				
	Missing	1 (4.3)	0	5 (5.7)				
Passive 6–10 yrs	Yes	16 (69.6)	24 (80.0)	70 (79.6)	0.03 (0.001–1.08)	0.055	0.04 (0.001–1.20)	0.064
	No	5 (21.7)	6 (20.0)	17 (19.3)				
	Missing	2 (8.7)	0	1 (1.1)				
Passive 11–18 yrs	Yes	18 (78.3)	26 (86.7)	72 (81.8)	2.70 (0.23–31.69)	0.429	1.06 (0.13–8.44)	0.957
	No	5 (21.7)	4 (13.3)	16 (18.2)				

Ileal (L1), Colonic (L2), Ileocolonic (L3).

*Adjusted for age, age of onset, gender, ethnicity, CD behavior at time of diagnosis and symptom duration before CD diagnosis.

^‡^Relative risk ratio, in comparison to risk of inflammatory disease.

^†^ Smoking during the 6 months prior to, or at time of diagnosis.

### Smoking Exposure and ‘Complicated’ Disease Behavior

On multiple logistic regression analysis no significant association was observed in patients who were smokers prior to, or at time of diagnosis and having ‘complicated’ CD (OR = 1.33; 95%CI, 0.60–2.92, p = 0.483) compared to non-smokers for complicated disease (Table 4). Furthermore, no significant risk-association was observed for complicated disease behavior at diagnosis and exposure to passive cigarette smoke during the 3 age intervals [(0–5 years: OR = 0.22; 95%CI, 0.03–1.52, p = 0.125); (6–10 years: OR = 1.92; 95%CI, 0.14–27.21, p = 0.629); and (11–18 years: OR = 0.91; 95%CI, 0.28–2.91, p = 0.872)] (Table 4). In addition, there was no significant association between cigarette smoking and complicated CD when non-smokers and smokers were analysed separately (Table 4).

**Table 4:** Cigarette smoking and complicated CD.

				Univariate analysis		Multivariate analysis*	
		Uncomplicated, n (%)	Complicated, n (%)	OR (95%CI)	P-value	OR (95%CI)	P-value
Overall							
Passive 0–5 yrs	Yes	83 (78.3)	61 (69.3)	0.64 (0.32–1.27)	0.200	0.22(0.03–1.52)	0.125
	No	20 (18.9)	23 (26.1)				
	Missing	3 (2.8)	4 (4.6)				
Passive 6–10 yrs	Yes	82 (77.4)	66 (75.0)	0.77 (0.39–1.52)	0.448	1.92 (0.14–27.21)	0.629
	No	21 (19.8)	22 (25.0)				
	Missing	3 (2.8)	0				
Passive 11–18 yrs	Yes	86 (81.1)	68 (77.3)	0.79 (0.39–1.59)	0.509	0.91 (0.28–2.91)	0.872
	No	20 (18.9)	20 (22.7)				
Smoking†	Yes	76 (71.7)	67 (76.1)	1.50 (0.76–2.98)	0.242	1.33 (0.60–2.92)	0.483
	No	29 (27.4)	17 (19.3)				
	Missing	1 (0.9)	4 (4.6)				
Non-smokers							
Passive 0–5 yrs	Yes	23 (79.3)	9 (52.9)	0.34(0.09–1.27)	0.109	0.28(0.06–1.36)	0.114
	No	6 (20.7)	7 (41.2)				
	Missing	0	1 (41.2)				
Passive 6–10 yrs	Yes	22 (75.9)	10 (58.8)	0.45 (0.13–1.65)	0.230	0.15 (0.01–1.98)	0.142
	No	7 (24.1)	7 (41.2)				
Passive 11–18 yrs	Yes	20 (69.0)	11 (64.7)	0.83 (0.23–2.93)	0.766	3.08 (0.22–42.91)	0.402
	No	9 (31.0)	6 (35.3)				
Smokers							
Passive 0–5 yrs	Yes	59 (77.6)	49 (73.1)	0.78(0.34–1.76)	0.543	0.38(0.05–3.12)	0.368
	No	14 (18.4)	15 (22.4)				
	Missing	3 (4.0)	3 (4.5)				
Passive 6–10 yrs	Yes	59 (77.5)	53 (79.1)	0.90 (0.39–2.06)	0.800	4.20 (0.42–41.58)	0.220
	No	14 (18.4)	14 (20.9)				
	Missing	3 (4.0)	0				
Passive 11–18 yrs	Yes	65 (85.5)	53 (79.1)	0.64 (0.27–1.53)	0.315	0.56 (0.12–2.51)	0.447
	No	11 (14.5)	14 (20.9)				

*Adjusted for gender, ethnicity, age at diagnosis, symptom duration before CD diagnosis and CD location at time of diagnosis.

^†^Smoking during 6 months prior to diagnosis, or at time of diagnosis.

## Discussion

A number of studies have identified active cigarette smoking as an independent risk factor for both the development and progression of CD [1,12,13,29,30], albeit such findings have not been consistently demonstrated, particularly among population groups outside of North America and Europe. Literature on the effect of passive cigarette smoke exposure and active cigarette smoking habits on the phenotypic outcomes of disease is limited, and in South Africa, no such data exists.

This study investigated the effect of active smoking habits and exposure to passive cigarette smoke during childhood on the phenotypic outcomes of CD at time of diagnosis, based on a cross sectional examination of all consecutive state-sector CD patients within the Western Cape, seen over a seventeen month period. Crohn’s disease patients who smoked during the 6 months prior to, or at time of diagnosis were significantly more likely to have either ileal (L1) or ileo-colonic (L3) disease at time of diagnosis, when compared to their non-smoking counterparts. Additionally, in smokers, passive cigarette smoke exposure during the 0–5 years age group was significantly associated with an increased risk for ileo-colonic CD.

The results of our study showed a strong association between smoking habits and both ileal (L1) and ileo-colonic (L3) disease at time of diagnosis. Similar associations have been previously reported [31–34], although such findings have not been consistently duplicated. For example, Lindberg et al. [35] found that cigarette was associated with ileal CD but that smoking did not influence colonic CD location, while Cosnes et al. [36], Tobin et al. [37] and Benoni et al. [38] all found no associations between cigarette smoking and CD location. Notably, these discrepancies may be attributed to the ‘broad’ definition used for disease location in different studies.

In this study we found that smoking habits prior to, or at the time of diagnosis were not associated with disease behavior. Our findings are similar to those obtained in a 2007 cross sectional study performed in Scotland by Aldhous et al., involving 408 CD patients. Similar findings were reported by Russell et al. in a prospective study in Holland [34], and in a case control study in the USA performed by Brant et al. [39]. However, in contrast to our results, other researchers have found that smoking was associated with aggressive CD phenotypes [4,36,40,41]. Differences in study designs, smoking definitions and study durations may account for some of the heterogeneity noted here.

The role of CD genetic susceptibility mutations on the effects of cigarette smoke on CD behaviour in different populations cannot be overlooked. For instance, in 2009, a Canadian study by Bhat et al, the authors found that, despite the higher prevalence of smoking among the French Canadian CD patients (n = 202), no significant difference in disease phenotype was observed, when compared to their Canadian counterparts (n = 1287) [42]. The authors concluded that this was because French Canadians have a higher genetic susceptibility to CD, which negated the effect of cigarette smoking. Of most interest however, are the findings of Leong et al., from a 2006 cross sectional study of 80 CD patients in China, which found cigarette smoking to be protective against the formation of granulomas (OR = 0.23; 95%CI, 0.07–0.75, p = 0.015), which are in turn commonly associated with stricturing disease behavior [18]. The authors suggested that suppression of TNFα and interleukin 1 (IL-1) and interleukin 6 (IL-6) by the nicotine in tobacco smoke may in turn suppress granuloma formation in the population studied. Notably, the three NOD2/CARD15 susceptibility genes commonly seen in the West are rare or absent among the Asian ethnicities [43] as well as in the South African Coloured population predominant in the Western Cape [23,25].

Apart from CD susceptibility genotype accounting for some these population-based discrepancies, it also remains plausible that the effect of cigarette smoking varies according to the stage of disease [1,19]. This makes inter-study comparisons difficult, for instance, the present study evaluated disease phenotype at time of diagnosis, whereas Leong et al. investigated the effect of cigarette smoking on granuloma formation in patients who had longstanding CD (median disease duration 4.1 years) [18]. In this study we analysed phenotype at the time of CD diagnosis, so we were not able to analyse the effect of disease duration on phenotype. Instead, we measured the time, in months, from the onset of symptoms to CD diagnosis, which was associated with ileal (p = 0.014) and ileo-colonic disease locations (p = 0.011) on univariate analysis, but not with disease behaviour. However, it is important to note that the true disease duration of CD may not be known as the biological time of disease onset is variable and may be impossible to ascertain.

Literature evaluating the effect of passive cigarette smoke exposure on the risk of future CD development has yielded inconsistent results [10,29,44–46]. For instance, in a retrospective study performed in 2009 by Van der Heide et al., the authors failed to identify an association between passive cigarette smoke exposure during childhood with either disease behavior, or disease location at the time of diagnosis in 380 CD patients, although immunosuppression therapy requirements were found to be significantly higher among those exposed [3]. In contrast, Basson et al. [S2 file] recently identified independent risk-association for passive smoke exposure during childhood (11–18 years) and risk of future CD development, in a South African cohort [27]. Childhood passive smoke exposure may be regarded as a form of low dose cigarette smoke exposure and at present, there is no firm data whether or not the influence of cigarette smoke exposure in CD etiopathogenesis is dose dependent. In the present study, childhood exposure to passive smoke during the 0–5 years age interval was associated with ileo-colonic disease in non-smokers and not associated with CD behavior.

One of the limitations of this study is the potential overlap in the childhood passive smoking age intervals, which means that that the passive exposure measured during the 0–5, 6–10 and 11–18 age intervals may not have been independent of each other. An analysis of cumulative exposure during at least two age intervals (data not shown) did not reveal any significant associations with either CD location or CD behaviour. Furthermore, in this research, we did not measure passive cigarette smoke exposure at the time of diagnosis, which may be associated with certain phenotypic features in non-smokers. Future studies could address this.

Another limitation of this study was the potential recall bias of childhood passive cigarette smoke exposure. In an attempt to minimize recall bias, the questionnaire consisted of predominantly multiple choice questions and after completion of the questionnaire with the interviewer, participants took the questionnaire home in order to consult family members on the accuracy of responses. The reliability of our data was evaluated via agreement analysis, as previously described [27]. In addition, smoking before diagnosis was verified by comparing the cases’ dates of CD diagnosis with their reported length of smoking. Finally, GSH and TBH are major referral centers, thus the identification of patients was hospital-based, and it is possible that our cohort underrepresented patients with a less severe CD. However the majority of patients using public health care services attend either GSH or TBH from where patients were recruited, and findings are likely generalizable.

## Conclusion

Cigarette smoking prior to, or at the time of CD diagnosis were associated with increased risk of ileal (L1) and ileo-colonic (L3) CD location. In smokers, childhood passive cigarette smoke exposure during the 0–5 years age interval may increase risk of later ileo-colonic CD. Future research is needed to support these findings, as well as establish whether cessation of smoking alters the clinical course of CD in our local setting.

## Supporting Information

S1 File Basson_Association btwn race and CD phenotype_2014. (PDF)

S2 File Basson_Association childhood environmental exposures and CD_2014. (PDF)
