# Correction: The Influence of Second-Hand Cigarette Smoke Exposure during Childhood and Active Cigarette Smoking on Crohn's Disease Phenotype Defined by the Montreal Classification Scheme in a Western Cape Population, South Africa

**DOI:** 10.1371/journal.pone.0190822

**Published:** 2018-01-02

**Authors:** Tawanda Chivese, Tonya M. Esterhuizen, Abigail Raffner Basson, Gillian Watermeyer

Dr. Gillian Watermeyer is not included in the author byline. She should be listed as the fourth author and affiliated with the Department of Gastroenterology, Groote Schuur Hospital, Cape Town, Western Cape, South Africa and the Department of Medicine, University of Cape Town, Cape Town, Western Cape, South Africa. The contributions of this author are as follows: conceptualization, data curation, funding acquisition, methodology, project administration, resources, supervision, validation.

The correct citation is: Chivese T, Esterhuizen TM, Basson AR, Watermeyer G (2015) The Influence of Second-Hand Cigarette Smoke Exposure during Childhood and Active Cigarette Smoking on Crohn’s Disease Phenotype Defined by the Montreal Classification Scheme in a Western Cape Population, South Africa. PLoS ONE 10(9): e0139597. https://doi.org/10.1371/journal.pone.0139597
