# Supplementary material for: The Influence of Second-Hand Cigarette Smoke Exposure during Childhood and Active Cigarette Smoking on Crohn’s Disease Phenotype Defined by the Montreal Classification Scheme in a Western Cape Population, South Africa
Source: PLoS One. 2015 Sep 30;10(9):e0139597. doi: 10.1371/journal.pone.0139597 (PMC4589355; doi:10.1371/journal.pone.0139597)
Supplement: S1 File — (PDF) [file pone.0139597.s001.pdf]

The Association between Race and Crohn's Disease Phenotype in the Western Cape Population of South Africa, Defined by the Montreal Classification System

Abigail Basson[1]*, Rina Swart[1], Esme Jordaan[2,3], Mikateko Mazinu[2], Gillian Watermeyer[4,5]

1 Dietetics Department, University of the Western Cape, Bellville, Western Cape, South Africa, 2 Biostatistics Unit, Medical Research Council of South Africa, Parow, Western Cape, South Africa, 3 Statistics and Population Studies Department, University of the Western Cape, Bellville, Western Cape, South Africa, 4 Department of Gastroenterology, Groote Schuur Hospital, Cape Town, Western Cape, South Africa, 5 Department of Medicine, University of Cape Town, Cape Town, Western Cape, South Africa

Abstract

Background: Inter-racial differences in disease characteristics and in the management of Crohn's disease (CD) have been described in African American and Asian subjects, however for the racial groups in South Africa, no such recent literature exists.

Methods: A cross sectional study of all consecutive CD patients seen at 2 large inflammatory bowel disease (IBD) referral centers in the Western Cape, South Africa between September 2011 and January 2013 was performed. Numerous demographic and clinical variables at diagnosis and date of study enrolment were identified using an investigator administered questionnaire as well as clinical examination and patient case notes. Using predefined definitions, disease behavior was stratified as 'complicated' or 'uncomplicated'.

Results: One hundred and ninety four CD subjects were identified; 35 (18%) were white, 152 (78%) were Cape Coloured and 7(4%) were black. On multiple logistic regression analysis Cape Coloureds were significantly more likely to develop 'complicated' CD (60% vs. 9%, $p=0.023$) during the disease course when compared to white subjects. In addition, significantly more white subjects had successfully discontinued cigarette smoking at study enrolment (31% vs. 7% reduction, $p=0.02$). No additional inter-racial differences were found. A low proportion of IBD family history was observed among the non-white subjects.

Conclusions: Cape Coloured patients were significantly more likely to develop 'complicated' CD over time when compared to whites.

Citation: Basson A, Swart R, Jordaan E, Mazinu M, Watermeyer G (2014) The Association between Race and Crohn's Disease Phenotype in the Western Cape Population of South Africa, Defined by the Montreal Classification System. PLoS ONE 9(8): e104859. doi:10.1371/journal.pone.0104859

Editor: Benoit Foligne, Institut Pasteur de Lille, France

Received March 18, 2014; **Accepted** July 18, 2014; **Published** August 12, 2014

Copyright: © 2014 Basson et al. This is an open-access article distributed under the terms of the Creative Commons Attribution License, which permits unrestricted use, distribution, and reproduction in any medium, provided the original author and source are credited.

Data Availability: The authors confirm that all data underlying the findings are fully available without restriction. Data published with Figshare. Basson, Abigail (2014): A.Basson_Crohn. figshare. http://dx.doi.org/10.6084/m9.figshare.1041586

Funding: This research was supported by the 2011 Scholarship in Gastroenterology; a grant from the AstraZeneca/South African Gastroenterology Society (SAGES). http://www.sages.co.za/D_AN_ScholTravel.asp. The funders had no role in the study design, data collection and analysis, decision to publish, or preparation of the manuscript.

Competing Interests: The authors have declared that no competing interests exist.

* Email: abbasson@uwc.ac.za

Introduction

A subtype of inflammatory bowel disease (IBD), Crohn's disease (CD) is believed to result from a complex interplay between genetic susceptibility and one or more environmental triggers. The disease is characterized as a chronic immune-mediated disorder of the gastrointestinal tract which may or may not be accompanied by a variety of extraintestinal or systemic complications. Disease presentation and severity are known to vary between individuals, having important implications for disease management [1,2]. Thus, in recent years, issues of CD sub-classification by phenotype have been reviewed, and the Montreal classification system [2] (revised Vienna) is the accepted standard.

Crohn's disease is found in all racial groups worldwide. However, historically, the highest prevalence rates have been reported in white populations, particularly those of North America and Europe, with significantly lower rates seen in black and Asian populations within these or any other foreign country [3–12]. As such, the majority of reports contributing to our understanding of disease presentation and clinical course originate primarily from Western populations, leaving a paucity of literature regarding the racial variability of CD phenotype [5,12–14].

Earlier epidemiological observations have suggested that CD presents in a more severe form in African American and Asian populations compared to their white counterparts [15–17], yet findings have been inconsistent, often limited by small sample size and variations in disease classification methods. Recently however,

reports indicate that the incidence of IBD in both African American and Asian populations has been steadily rising over the decades [18–22]. This increase in incidence may be attributed to one of several factors namely, changes in utilization and accessibility of hospital care (suggesting an underreporting of previous incidence rates), selection bias of IBD centers, or a true rise in the incidence among these racial groups [23]. Therefore, aspects surrounding the racial variations in CD phenotype should continue to be explored and include all non-white populations in order to further contribute towards our understanding of the environmental and genetic factors involved in the disease etiology.

The aim of our study was thus to provide a preliminary and descriptive view on disease phenotype of the racial groups in Cape Town, South Africa.

Materials and Methods

The study protocol and questionnaire were reviewed and approved by the Senate Research Ethics Committee of the University of the Western Cape (Reg no. 11/3/16), the Human Research Ethics Committee of the University of Cape Town (HREC REF: 122/2011) and the Provincial Department of Health. All participants gave written informed consent. http://dx.doi.org/10.6084/m9.figshare.1041586.

Design and Setting

This was a cross sectional examination (part of a larger case-control study) of all consecutive white, Cape Coloured and black CD patients seen at Groote Schuur Hospital (GSH) and Tygerberg Hospital (TBH) during normally scheduled appointments between September 2011 and January 2013. GSH and TBH manage all public-sector IBD patients within the Western Cape, South Africa. Of the 3.5 million persons who reside in the greater Cape Town area, approximately 90% rely on the public-sector health services [24]. Cape Coloureds are subjects of mixed-ancestry. The term which is non-derogatory refers to a heterogeneous ethnic group of which genome analysis has now identified South Asian, European, Indonesian and isiXhosa sub-Saharan blacks as the four predominant genetic contributors [25]. Disease diagnosis was defined by the European Crohn's and Colitis Organization (ECCO) guidelines [26].

Following informed consent, data relating to patient demographics, smoking and disease symptoms prior to diagnosis were collected via an interviewer administered questionnaire. Race was self-reported. Information relating to disease characteristics and disease course were determined via review of patient medical records as well as clinical examination by the consulting gastroenterologist. Monthly income was determined using computerized hospital records. Only patients with complete data at diagnosis were included. Patients were excluded if disease duration was less than 5 years, or had a prior diagnosis of intestinal tuberculosis. In accordance with the paper published by Epstein et al. [27] there is no gold standard in the differential diagnosis between CD and intestinal tuberculosis however as per the algorithm suggested by these authors every attempt was made to exclude a diagnosis of tuberculosis.

The Montreal classification system [2] (Table 1) was used to define age of onset, disease location and disease behavior at two time intervals; initial diagnosis and date of study enrolment. Any disease related surgical history was categorized by timing of first surgery in relation to initial diagnosis. Information on medical management included lifetime use of immunomodulator, anti-tumor necrosis factor inhibitors or 5-aminosalicylates. Complicated disease was defined as the presence of any one of the following at diagnosis or during subsequent follow ups: stricturing CD, penetrating CD, perianal fistulas or surgical resection. Data on extraintestinal manifestations (EIMs) was divided into four categories: (1) skin, (2) ocular, (3) joint and (4) other. Skin manifestations included erythema nodosum, pyoderma gangrenosum, and neutrophilic dermatoses. Ocular manifestations included uveitis, iritis, and episcleritis. Joint manifestations included arthralgias, arthritis and axial arthropathies. The 'other' manifestations included ankylosing spondylitis and primary biliary cirrhosis.

Data Analysis

Descriptive data is presented overall as well as separately for the three racial groups (white, Cape Coloured and black) as medians (IQRs) for numerical data, and as frequencies and percentages for categorical data. The Kruskall-Wallis test was used to compare racial groups with respect to their medians for the numeric demographic variables and the Fisher's exact test was used to compare the percentages for the categorical variables. All statistical analysis included only white and Cape Coloured subjects, due to the small number of black subjects. A separate multiple logistic regression model was conducted for each phenotype (age of onset, disease location and disease behavior) to test for an association between the phenotype and racial groups (whites and Cape Coloureds), adjusting for possible confounders age of onset, gender, smoking and duration of symptom onset as appropriate. Separate contingency tables for medication use, surgical interventions and EIMs versus racial group were conducted and the Fisher's exact test was used to test for associations. No adjustments were made in the latter analysis. A generalized linear model (GEE with an unstructured correlation matrix) was used to test for an interaction between racial groups and smoking and phonotype from diagnosis to study enrolment. The standard for significance for all analysis was $P<0.05$.

Results

Demographic Characteristics of Subjects

Over an approximate seventeen month period, 194 CD patients meeting our inclusion criteria were identified and consented to the study, 35 (18%) were white, 152 (78%) were Cape Coloured and 7 (4%) were black. Two patients that were approached declined to participate (response rate = 99%). Demographic and baseline characteristics for each racial group are shown in Table 2. Overall, 9 (26%) white, 41 (27%) Cape Coloured and 3 (43%) black subjects were male. The median age at enrolment was 47.0 (IQR 38–57) years, the median age of disease onset was 28 (IQR 21.5–38.0) years and median disease duration was 16 (IQR 10.0–24.0) years. The majority of subjects in all racial groups were born in South Africa (95%), but individually, 100% of the Cape Coloureds compared to 77% of the white subjects, were born in South Africa ($p<0.001$). There was no significant difference in the level of education between the white and Cape Coloured subjects, however there was a significant difference in the median age at study enrolment [52.0 (IQR 40.0–67.5) years vs. 46.0 (IQR 38.0–55.5) years, respectively] as whites were on average six years older at study enrolment ($p = 0.04$). Comparing white subjects with their Cape Coloured counterparts, median disease duration [22.0 (IQR 10.5–25.0) years vs. 16.0 (IQR 10.0–24.0) years] and median duration of initial presenting symptoms [2.0 (IQR 0.6–3.5) years vs. 1.0 (IQR 0.5–3.0) years], appeared longer in the white subjects, although results did not reach statistical significance. No significant inter-racial difference was found in the smoking habits for white and Cape Coloured subjects at diagnosis (74% vs. 63%). When

Table 1. Montreal Classification Scheme.

Age at diagnosis (years)	
A1	≤16
A2	17–40
A3	>40
Disease location	
L1	Isolated to the terminal ileum
L2	Isolated to the colon
L3	Ileum and colonic involvement
L4*	Upper gastrointestinal tract
Disease behavior	
B1†	Inflammatory; non-stricturing, non-penetrating
B2	Stricturing
B3	Penetrating disease, with or without stricturing, excludes perianal penetrating disease
p‡	Perianal disease modifier

*Upper gastrointestinal (GI) modifier (L4) can be added to L1–L3 when concomitant upper GI disease present.
†B1 category should be considered "interim" until a pre-specified time has elapsed from time of diagnosis. Suggested time period is between 5–10 years.
‡"p" is added to B1–B3 when concomitant perianal disease is present.
doi:10.1371/journal.pone.0104859.t001

comparing the change in smoking habits from time of diagnosis to study enrolment for the racial groups, we found a significant interaction (Chi-Square = 5.4; p = 0.02), and the results indicated that the reduction of 31% smoking for whites was significant (p = 0.001), but the reduction of 6.8% Cape Coloureds smoking was not significant (p = 0.08).

Disease Characteristics between Racial Groups

On multiple logistic regression analysis there was no significant difference between Cape Coloured and white subjects with regards to disease location or disease behavior at diagnosis (p>0.05; Table 3). However after a median disease duration of 16 years, significantly more Cape Coloured subjects had developed 'complicated' CD (60% vs. 9%, p = 0.023) during the disease course

Table 2. Demographic and Baseline Characteristics of Patients.

		White (*n* = 35)	Cape Coloured (*n* = 152)	Black (*n* = 7)	Overall (*N* = 194)	p-value*
Gender, no. (%)	Males	9 (26)	41 (27)	3 (43)	53 (27)	0.88
	Females	26 (74)	111 (73)	4 (57)	141 (73)	
Age at enrolment (median and IQR), yr.†		52.0 (40.0, 67.5)	46.0 (38.0, 55.5)	44.0 (38.0, 46.0)	47 (38.0, 57.0)	0.04
Age at diagnosis (median and IQR), yr.		28.0 (21.5, 45.5)	28.5 (21.5, 45.5)	29.0 (26.5, 33.5)	28 (21.5, 38.0)	0.24
Disease duration (median and IQR), yr.		22.0 (10.5, 25.0)	16.0 (10.0, 24.0)	12.0 (9.5, 14.0)	16 (10.0, 24.0)	0.30
Duration presenting symptoms (median and IQR), yr.		2.0 (0.6, 3.5)	1.0 (0.5, 3.0)	1.0 (0.2, 1.0)	1.0 (0.5, 3.5)	0.58
Married, no. (%)‡		14 (40)	78 (51)	1 (14)	93 (48)	0.64
Education, no. (%)§		13 (37)	21 (14)	2 (29)	36 (18)	0.12
Born in South Africa, no. (%)		27 (77)	152 (100)	5 (71)	184 (95)	<0.001
Income per month, no. (%)						
	<R3000	30 (86)	127 (84)	6 (86)	163 (84)	0.74
	R3000–10,000	4 (11)	23 (15)	1 (14)	28 (14)	
	>R10,000	1 (3)	2 (1)	0 (0)	3 (1)	
Smoking history, no. (%)[a]	At diagnosis	26 (74)	95 (63)	6 (86)	127 (65)	0.42
	At study enrolment	15 (43)	87 (57)	2 (29)	104 (54)	0.09
Family History IBD, no. (%)[b]		5 (14)	10 (6)	0 (0)	15 (7)	0.75

IQR, interquartile range; IBD, inflammatory bowel disease.
*Statistical analysis excluding black subjects. No subjects reported being of Indian or Asian ethnicity.
†Age at study enrolment missing for 1 Cape Coloured subject.
‡Civil marriage or living with a partner.
§At least some tertiary education.
[a]Smoking status at diagnosis; data missing for 5 subjects. Smoking status at study enrolment; data missing for 7 subjects.
[b]Family history IBD defined as parents, siblings or offspring.
doi:10.1371/journal.pone.0104859.t002

Table 3. Patient Phenotype According to Montreal Classification Scheme.

		Diagnosis					Study enrolment				
		White (n=35)	Cape Coloured (n=152)	Black (n=7)	Overall (N=194)	p- value*	White (n=35)	Cape Coloured (n=152)	Black (n=7)	Overall (N=194)	p- value*
Age at diagnosis and at enrolment, no. (%)[a]	A1	1 (3)	15 (10)	1 (14)	17 (9)	0.43†	0 (0)	0 (0)	0 (0)	0 (0)	0.84
	A2	22 (63)	113 (74)	5 (71)	140 (72)		9 (26)	50 (33)	3 (43)	62 (31)	
	A3	12 (37)	24 (16)	1 (14)	37 (19)		26 (74)	101 (67)	4 (57)	131 (68)	
Disease location, no. (%)[b]	L1	11 (31)	27 (18)	1 (14)	39 (20)	0.65‡	13 (37)	50 (33)	2 (29)	65 (33)	0.87§
	L2	6 (17)	30 (20)	1 (14)	37 (19)		10 (28)	39 (26)	0 (0)	49 (26)	
	L3	18 (52)	93 (61)	5 (72)	116 (61)		12 (34)	62 (41)	5 (71)	79 (41)	
Disease behavior, no. (%)	B1	21 (60)	81 (53)	3 (43)	105 (54)	0.21‡	21 (60)	76 (50)	2 (29)	99 (51)	0.18§
	B2	5 (14)	36 (24)	1 (14)	42 (22)		6 (17)	39 (26)	1 (14)	46 (24)	
	B3	9 (26)	35 (21)	3 (43)	47 (24)		8 (23)	37 (24)	4 (57)	49 (25)	
	p[c]	9 (26)	36 (24)	3 (43)	48 (25)	0.73‡	3 (9)	20 (13)	2 (29)	25 (13)	0.39§

*Statistical analysis excludes black patients.
†Adjusted for gender, smoking and duration of symptom onset.
‡Adjusted for gender, smoking, age of onset and duration of symptom onset.
§Adjusted for gender, smoking, disease duration and age of onset (as appropriate).
[a]Age at study enrolment; data missing for 1 Cape Coloured subject.
[b]Disease location was not confirmed by upper endoscopy, ileocolonoscopy or small bowel imaging for 2 Cape Coloured subject at diagnosis and 1 Cape Coloured subject at study enrolment. Overall, no subjects had upper gastrointestinal disease
[c]"p" is the perianal modifier added to B1–B3 when concomitant perianal disease is present.
doi:10.1371/journal.pone.0104859.t003

Table 4. Patient Medical and Surgical Management and Extraintestinal Manifestations (EIMs).

		White (*n* = 35)	Cape Coloured (*n* = 152)	Black (*n* = 7)	Overall (*n* = 194)	p-value*
Medication use over disease course, no. (%)†	Corticosteroids	6 (17)	32 (21)	1 (14)	39 (20)	0.82
	5-aminosalicylates	10 (29)	60 (40)	0 (0)	70 (36)	0.32
	Immunomodulators	19 (54)	109 (72)	4 (57)	132 (68)	0.48
	Tumor necrosis factor inhibitors	1 (3)	8 (5)	0 (0)	9 (5)	1.00
Lifetime surgical intervention, no. (%)		14 (40)	86 (57)	4 (57)	104 (54)	0.16
Timing of first surgery after diagnosis, no. (%)‡	Within 1 year	6 (17)	44 (29)	2 (29)	52 (27)	0.84
	Within 1–5 years	2 (6)	17 (11)	1 (14)	20 (10)	
	After 5 years	4 (11)	21 (14)	1 (14)	26 (13)	
Type of EIM over disease course, no. (%)	Skin	3 (9)	13 (9)	2 (29)	18 (93)	1.00
	Ocular	0 (0)	2 (1)	1 (14)	3 (2)	1.00
	Joint	10 (29)	46 (30)	3 (43)	59 (30)	0.37
	Other§	1 (3)	2 (1)	0 (0)	3 (2)	0.46

*Statistical analysis excludes black patients.
†Excludes 15 patients with insufficient records of medical management.
‡Excludes 12 patients with incomplete records of surgical dates.
§Other EIM disorders included; ankylosing spondylitis and primary biliary cirrhosis.
doi:10.1371/journal.pone.0104859.t004

when compared to whites (results not shown). There was however no significant inter-racial difference in terms of EIMs, surgical or medical management over disease course (Table 4).

Although the numbers were too small for meaningful analysis all black subjects developed complicated CD within a mean of 1.71 (SD±1.25) years after initial diagnosis. In addition 43% already had a penetrating disease phenotype or perianal fistulas at diagnosis. None of the black subjects reported a first degree family history of IBD, compared to 6% of the Cape Coloured and 14% of the white patients. Overall, less than 10% of patients had a family history of IBD.

Discussion

Population based epidemiological studies remain of paramount importance in piecing together the complex pathogenesis underlying CD, particularly in terms of inter-racial variations of disease phenotype. In South Africa, while a number of earlier reports are available, no such recent data exists for the population; a population broadly classified into three ethnic groups: black south African, white and Cape Coloured.

This study included all consecutive state-sector adult CD patients within the Western Cape, South Africa seen over a seventeen month period. Comparing Cape Coloured subjects with their white counterparts, a significant difference in the development of 'complicated' CD (60% vs. 9%) over time was noted. One possible explanation is the high prevalence of ongoing active smoking in Cape Coloureds at study enrolment. Cigarette smoking is a well described risk factor for the development of complicated and aggressive CD over time [28]. The rate of complicated CD in our white subjects was lower compared to that described in other populations. It is possible that the discrepancy in disease course can be attributed to differences in treatment strategies, patient compliance to medical management, or true microbial differences between populations, but these factors were not investigated. In addition, recent reports indicate that prevalence of systemic lupus erythematosus, particularly among the black and Coloured females in Cape Town, South Africa is higher than previously thought [29,30]. However in this present study, no concomitant diagnosis of CD and systemic lupus erythematosus was found, suggesting that different factors contribute to the etiology of the two diseases.

In keeping with reports from Asia [31–44], among our Cape Coloured subjects, ileo-colonic appeared to be the most common location at diagnosis. Interestingly in contrast to white patients, Cape Coloured subjects appeared to have a shorter duration of presenting symptoms in years until diagnosis. These findings are at odds with the widely held belief that the disease is frequently overlooked in this population due to the high rates of tuberculosis and infectious diarrhea, or poor access to health care. Notably, the public-sector health care system in South Africa predominantly caters to those who are of lower socioeconomic standing. In this study, findings are likely not attributed to inter-racial differences in access to healthcare or medical treatment as there was no significant inter-racial difference in the level of income. Moreover education is considered to be a good marker of socioeconomic status and in our cohort, there was no significant inter-racial difference in the level of education.

Family history of IBD is considered one of the strongest predisposing risk factors in CD. However in our cohort 6% of the Cape Coloured and none of the black patients reported having a first degree family member with IBD. In contrast 14% of the white subjects had a positive family history, the latter compatible with Western data (10–25%) [43,45,46]. This finding may reflect racial differences in CD susceptibility mutations. A study of South African Coloureds failed to demonstrate an association with 3 nucleotide oligomerization domain (NOD-2) mutations commonly seen in the West [47]. Moreover, significant differences of allele and genotype frequencies in the -237C→T promoter polymorphisms of the *SCL11A1* gene, a gene implicated in CD susceptibility [48], were observed in South African Coloured CD patients, but not in their white and black counterparts [49]. Similar discrepancies have been observed in Japanese [50,51], Han Chinese [52–54], Korean [55], Indian [56] and Malaysian [57] populations [58,59].

A five year retrospective study [60] based on the GSH gastrointestinal clinic patient lists, found an increasing CD

incidence in the Coloured population; from 0.4/100 000 per year during 1970–1974 [61] to 1.3/100 000 per year during 1975–1980. Of the 117 CD patients reviewed; 32 were Coloured and only 1 was black. In contrast, we found 152 Cape Coloured and 7 black, consecutive CD state sector patients. Given the significant socioeconomic, dietary and lifestyle changes that have taken place over the past two decades, it is likely that our findings indicate an epidemiological transition among these racial groups, a trend noted in developed and developing countries alike [5,18]. These observed trends in our Cape Coloured and black subjects lend support to both different susceptibility genes and variable environmental interactions between racial groups, implying distinctions in disease pathogenesis or risk.

Our study was limited by the small number of black subjects. It is possible that over a longer period, including patients from both, the state and private sector, a larger sample size would be drawn. Data regarding medication use has been poorly captured in the past, as this was a retrospective study, details on the type medical treatment used, duration of treatment, dosage and adherence was difficult to determine, and may have contributed to the identified inter-racial differences. Similarly, given the retrospective nature of the study, the exact parameters used to exclude tuberculosis diagnosis (i.e., histological, radiological, endoscopic), were not available to report in this paper. Our cohort may have included a higher proportion of patients with 'complicated' disease as GSH and TBH are both referral based IBD centers. Poor socioeconomic status is associated with helminth infection and in South Africa helminth infection has been shown to be protective against IBD development [62]. Therefore it is entirely possible that within our cohort this may have influenced the severity of CD between the racial groups however this was not one of the variables evaluated in the present study. The ethnic diversity of our cohort was also not representative to that of the Western Cape, as 2011 provincial estimates approximate (N = 5,822,734) 15.7% of the population as white, 48.8% as Colored and 32.9% as black (excluding Indian and Asian ethnicities) [63]. We also did not verify the self-reported race using genetic markers. However, validity of self-reported racial status has been previously acknowledged, as very low rates of discordance between self-reported racial status and genetic markers have been described [25,64,65].

Acknowledgments

We thank the Raffners, Dr. Ernesta Kunneke, Karin Fenton, Ushma Galal and Amanda Fourie for their inspiring and administrative assistance of this research

Author Contributions

Conceived and designed the experiments: AB RS GW. Performed the experiments: AB. Analyzed the data: EJ MM AB. Contributed reagents/materials/analysis tools: EJ. Contributed to the writing of the manuscript: AB RS GW EJ MM. Critical Revision manuscript: AB RS GW EJ MM. Data Interpretation: EJ MM.

References

1. Freeman Hj (2009) Long-Term Natural History Of Crohn's Disease. World J Gastroenterol 15: 1315–1318.
2. Silverberg Ms, Satsangi J, Ahmad T, Arnott I, Bernstein Cn, et al. (2005) Toward An Integrated Clinical, Molecular And Serological Classification Of Inflammatory Bowel Disease: Report Of A Working Party Of The 2005 Montreal World Congress Of Gastroenterology. Can J Gastroenterol 19: 5–36.
3. Straus Wl, Eisen Gm, Sandler Rs, Murray Sc, Sessions Jt (2000) Crohn's Disease: Does Race Matter? Am J Gastroenterol 95: 479–483.
4. Karlinger K, Györke T, Makö E, Mester Á, Tarján Z (2000) The Epidemiology And The Pathogenesis Of Inflammatory Bowel Disease. Eur J Radiol 35: 154–167.
5. Loftus Ev, Sandborn Wj (2002) Epidemiology Of Inflammatory Bowel Disease. Gastroenterol Clin North Am 31: 1–20.
6. Rogers B, Clark Lm, Kirsner Jb (1971) The Epidemiologic And Demographic Characteristics Of Inflammatory Bowel Disease: An Analysis Of A Computerized File Of 1400 Patients. J Chronic Dis 24: 743–773.
7. Mendeloff Ai, Monk M, Siegel Ci, Lilienfeld A (1966) Some Epidemiological Features Of Ulcerative Colitis And Regional Enteritis. A Preliminary Report. Gastroenterology 51: 748–756.
8. Samuels Ad, Weese Jl, Berman Pm, Kirsner Jb (1974) An Epidemiologic And Demographic Study Of Inflammatory Bowel Disease In Black Patients. Am J Dig Dis 19: 156–160.
9. Mosley Jr E, Rogers N, Scott V, Chung E, Press Jr Hc, et al. (1977) Crohn's Disease In Black Patients. J Natl Med Assoc 69: 219–222.
10. Mekhjian H, Switz D, Melnyk C, Rankin G, Brooks R (1979) Clinical Features And Natural History Of Crohn's Disease. Gastroenterology 77: 898–906.
11. Simsek H, Schuman Bm (1989) Inflammatory Bowel Disease In 64 Black Patients: Analysis Of Course, Complications, And Surgery. J Clin Gastroenterol 11: 294–298.
12. Loftus Ev (2004) Clinical Epidemiology Of Inflammatory Bowel Disease: Incidence, Prevalence, And Environmental Influences. Gastroenterology 126: 1504–1517.
13. Loftus Ev, Silverstein Md, Sandborn Wj, Tremaine Wj, Harmsen Ws, et al. (1998) Crohn's Disease In Olmsted County, Minnesota, 1940–1993: Incidence, Prevalence, And Survival. Gastroenterology 114: 1161–1168.
14. Vind I, Riis L, Jess T, Knudsen E, Pedersen N, et al. (2006) Increasing Incidences Of Inflammatory Bowel Disease And Decreasing Surgery Rates In Copenhagen City And County, 2003–2005: A Population-Based Study From The Danish Crohn Colitis Database. Am J Gastroenterol 101: 1274–1282.
15. Thia Ktj, Luman W, Jin Oc (2006) Crohn's Disease Runs A More Aggressive Course In Young Asian Patients. Inflamm Bowel Dis 12: 57–61.
16. Goldman Cd, Kodner Ij, Fry Rd, Macdermott Rp (1986) Clinical And Operative Experience With Non-Caucasian Patients With Crohn's Disease. Dis Colon Rectum 29: 317–321.
17. Paul Jr H, Barnes R, Reese Ve, Childress Mh, Scott V, et al. (1990) Crohn's Disease In Black Patients. J Natl Med Assoc 82: 709–712.
18. Molodecky Na, Soon Is, Rabi Dm, Ghali Wa, Ferris M, et al. (2012) Increasing Incidence And Prevalence Of The Inflammatory Bowel Diseases With Time, Based On Systematic Review. Gastroenterology 142: 46–54. E42.
19. Calkins Bm (1984) Trends In Incidence Rates Of Ulcerative Colitis And Crohn's Disease. Dig Dis Sci 29: 913–920.
20. Kurata Jh, Kantor-Fish S, Frankl H, Godby P, Vadheim Cm (1992) Crohn's Disease Among Ethnic Groups In A Large Health Maintenance Organization. Gastroenterology 102: 1940–1948.
21. Hou Jk, El-Serag H, Thirumurthi S (2009) Distribution And Manifestations Of Inflammatory Bowel Disease In Asians, Hispanics, And African Americans: A Systematic Review. Am J Gastroenterol 104: 2100–2109.
22. Thia Kt, Loftus Ev, Sandborn Wj, Yang S (2008) An Update On The Epidemiology Of Inflammatory Bowel Disease In Asia. Am J Gastroenterol 103: 3167–3182.
23. Basu D, Lopez I, Kulkarni A, Sellin Jh (2005) Impact Of Race And Ethnicity On Inflammatory Bowel Disease. Am J Gastroenterol 100: 2254–2261.
24. Small K (2008) Demographic And Socio-Economic Trends For Cape Town: 1996 To 2007. Unpublished Report, City Of Cape Town.
25. Patterson N, Petersen Dc, Van Der Ross Re, Sudoyo H, Glashoff Rh, et al. (2010) Genetic Structure Of A Unique Admixed Population: Implications For Medical Research. Hum Mol Genet 19: 411–419.
26. Stange E, Travis S, Vermeire S, Beglinger C, Kupcinkas L, et al. (2006) European Evidence Based Consensus On The Diagnosis And Management Of Crohn's Disease: Definitions And Diagnosis. Gut 55: I1–I15.
27. Epstein D, Watermeyer G, Kirsch R (2007) Review Article: The Diagnosis And Management Of Crohn's Disease In Populations With High-Risk Rates For Tuberculosis. Aliment Pharmacol Ther 25: 1373–1388.
28. Aldhous Mc, Satsangi J (2010) The Impact Of Smoking In Crohn's Disease: No Smoke Without Fire. Frontline Gastroenterol 1: 156–164.
29. Ayodele Oe, Okpechi Ig, Swanepoel Cr (2010) Predictors Of Poor Renal Outcome In Patients With Biopsy-Proven Lupus Nephritis. Nephrology 15: 482–490.
30. Wadee S, Tikly M, Hopley M (2007) Causes And Predictors Of Death In South Africans With Systemic Lupus Erythematosus. Rheumatology (Oxford) 46: 1487–1491.
31. Shin Dh, Sinn Dh, Kim Y, Kim Jy, Chang Dk, et al. (2011) Increasing Incidence Of Inflammatory Bowel Disease Among Young Men In Korea Between 2003 And 2008. Dig Dis Sci 56: 1154–1159.
32. Ishige T, Tomomasa T, Takebayashi T, Asakura K, Watanabe M, et al. (2010) Inflammatory Bowel Disease In Children: Epidemiological Analysis Of The Nationwide Ibd Registry In Japan. J Gastroenterol 45: 911–917.

33. Jiang L, Xia B, Li J, Ye M, Yan W, et al. (2006) Retrospective Survey Of 452 Patients With Inflammatory Bowel Disease In Wuhan City, Central China. Inflamm Bowel Dis 12: 212–217.
34. Song X, Gao X, Li M, Chen Z, Chen S, et al. (2011) Clinical Features And Risk Factors For Primary Surgery In 205 Patients With Crohn's Disease: Analysis Of A South China Cohort. Dis Colon Rectum 54: 1147–1154.
35. Cao Q, Si J, Gao M, Zhou G, Hu W, et al. (2005) Clinical Presentation Of Inflammatory Bowel Disease: A Hospital Based Retrospective Study Of 379 Patients In Eastern China. Chin Med J 118: 747–752.
36. Chow Dk, Leong Rw, Lai Lh, Wong Gl, Leung W, et al. (2008) Changes In Crohn's Disease Phenotype Over Time In The Chinese Population: Validation Of The Montreal Classification System. Inflamm Bowel Dis 14: 536–541.
37. Oriuchi T, Hiwatashi N, Kinouchi Y, Takahashi S, Takagi S, et al. (2003) Clinical Course And Longterm Prognosis Of Japanese Patients With Crohn's Disease: Predictive Factors, Rates Of Operation, And Mortality. J Gastroenterol 38: 942–953.
38. Ye Bd, Yang S, Cho Yk, Park Sh, Yang D, et al. (2010) Clinical Features And Long-Term Prognosis Of Crohn's Disease In Korea. Scand J Gastroenterol 45: 1178–1185.
39. Lawrance Ic, Murray K, Hall A, Sung Jj, Leong R (2004) A Prospective Comparative Study Of Asca And Panca In Chinese And Caucasian Ibd Patients. Am J Gastroenterol 99: 2186–2194.
40. Hisabe T, Matsui T, Sakurai T, Murakami Y, Tanabe H, et al. (2003) Anti-Saccharomyces Cerevisiae Antibodies In Japanese Patients With Inflammatory Bowel Disease: Diagnostic Accuracy And Clinical Value. J Gastroenterol 38: 121–126.
41. Leong Rw, Lawrance Ic, Chow Dk, To K, Lau Jy, et al. (2006) Association Of Intestinal Granulomas With Smoking, Phenotype, And Serology In Chinese Patients With Crohn's Disease. Am J Gastroenterol 101: 1024–1029.
42. Chow Dk, Sung Jj, Wu Jc, Tsoi Kk, Leong Rw, et al. (2009) Upper Gastrointestinal Tract Phenotype Of Crohn's Disease Is Associated With Early Surgery And Further Hospitalization. Inflamm Bowel Dis 15: 551–557.
43. Orholm M, Munkholm P, Langholz E, Nielsen Oh, Sφrensen Ti, et al. (1991) Familial Occurrence Of Inflammatory Bowel Disease. N Engl J Med 324: 84–88.
44. Yang S, Yun S, Kim J, Park Jy, Kim Hy, et al. (2008) Epidemiology Of Inflammatory Bowel Disease In The Songpa-Kangdong District, Seoul, Korea, 1986–2005: A Kasid Study. Inflamm Bowel Dis 14: 542–549.
45. Peeters M, Nevens H, Baert F, Hiele M, De Meyer Am, et al. (1996) Familial Aggregation In Crohn's Disease: Increased Age-Adjusted Risk And Concordance In Clinical Characteristics. Gastroenterology 111: 597–603.
46. Bayless T, Tokayer A, Polito J, Quaskey S, Mellits E, et al. (1996) Crohn's Disease: Concordance For Site And Clinical Type In Affected Family Members-Potential Hereditary Influences. Gastroenterology 111: 573–579.
47. Zaahl Mg, Winter Ta, Warnich L, Kotze Mj (2005) Analysis Of The Three Common Mutations In The Card15 Gene (R702w, G908r And 1007fs) In South African Colored Patients With Inflammatory Bowel Disease. Mol Cell Probes 19: 278–281.
48. Hofmeister A, Neibergs Hl, Pokorny Rm, Galandiuk S (1997) The Natural Resistance-Associated Macrophage Protein Gene Is Associated With Crohn's Disease. Surgery 122: 173–179.
49. Zaahl Mg, Winter Ta, Warnich L, Kotze Mj (2006) The -237c—>T Promoter Polymorphism Of The Slc11a1 Gene Is Associated With A Protective Effect In Relation To Inflammatory Bowel Disease In The South African Population. Int J Colorectal Dis 21: 402–408.
50. Inoue N, Tamura K, Kinouchi Y, Fukuda Y, Takahashi S, et al. (2002) Lack Of Common Nod2 Variants In Japanese Patients With Crohn's Disease. Gastroenterology 123: 86–91.
51. Yamazaki K, Takazoe M, Tanaka T, Kazumori T, Nakamura Y (2002) Absence Of Mutation In The Nod2/Card15 Gene Among 483 Japanese Patients With Crohn's Disease. J Hum Genet 47: 469–472.
52. Guo Q, Xia B, Jiang Y, Qu Y, Li J (2004) Nod2 3020insc Frameshift Mutation Is Not Associated With Inflammatory Bowel Disease In Chinese Patients Of Han Nationality. World J Gastroenterol 10: 1069–1071.
53. Leong R, Armuzzi A, Ahmad T, Wong M, Tse P, et al. (2003) Nod2/Card15 Gene Polymorphisms And Crohn's Disease In The Chinese Population. Aliment Pharmacol Ther 17: 1465–1470.
54. Li M, Gao X, Guo C, Wu K, Zhang X, et al. (2008) Octn And Card15 Gene Polymorphism In Chinese Patients With Inflammatory Bowel Disease. World J Gastroenterol 14: 4923–4927.
55. Croucher Pj, Mascheretti S, Hampe J, Huse K, Frenzel H, et al. (2003) Haplotype Structure And Association To Crohn's Disease Of Card15 Mutations In Two Ethnically Divergent Populations. Eur J Hum Genet 11: 6–16.
56. Pugazhendhi S, Amte A, Balamurugan R, Subramanian V, Ramakrishna Bs (2008) Common Nod2 Mutations Are Absent In Patients With Crohn's Disease In India. Indian J Gastroenterol 27: 201–203.
57. Chua Kh, Hilmi I, Ng Cc, Eng Tl, Palaniappan S, et al. (2009) Identification Of Nod2/Card15 Mutations In Malaysian Patients With Crohn's Disease. J Dig Dis 10: 124–130.
58. Yamazaki K, Mcgovern D, Ragoussis J, Paolucci M, Butler H, et al. (2005) Single Nucleotide Polymorphisms In Tnfsf15 Confer Susceptibility To Crohn's Disease. Hum Mol Genet 14: 3499–3506.
59. Yang S, Lim J, Chang H, Lee I, Li Y, et al. (2008) Association Of Tnfsf15 With Crohn's Disease In Koreans. Am J Gastroenterol 103: 1437–1442.
60. Wright Jp, Marks In, Jameson C, Garisch Ja, Burns Dg, et al. (1983) Inflammatory Bowel Disease In Cape Town, 1975–1980. Part Ii. Crohn's Disease. S Afr Med J 63: 226–229.
61. Novis Bh, Marks In, Bank S, Louw Jh (1975) Incidence Of Crohn's Disease At Groote Schuur Hospital During 1970–1974. S Afr Med J 49: 693–697.
62. Chu Km, Watermeyer G, Shelly L, Janssen J, May Td, et al. (2013) Childhood Helminth Exposure Is Protective Against Inflammatory Bowel Disease: A Case Control Study In South Africa. Inflamm Bowel Dis 19: 614–620.
63. Statistics South Africa (October 30, 2012) Census 2011: Statistical Release (Revised) P0301.4. 2013.
64. Risch N (2006) Dissecting Racial And Ethnic Differences. N Engl J Med 354: 408–411.
65. Tang H, Quertermous T, Rodriguez B, Kardia Sl, Zhu X, et al. (2005) Genetic Structure, Self-Identified Race/Ethnicity, And Confounding In Case-Control Association Studies. Am J Hum Genet 76: 268–275.
